# Microbiology of Bartholin's Duct Abscess

**DOI:** 10.1155/S1064744994000220

**Published:** 1994

**Authors:** Anu Mattila, Ari Miettinen, Pentti K. Heinonen

**Affiliations:** 1 Department of Clinical Microbiology Tampere University Hospital P.O. Box 2000 Tampere FIN-33521 Finland; 2 Department of Obstetrics and Gynecology University of Tampere Tampere Finland; 3 Department of Clinical Medicine University of Tampere Tampere Finland

## Abstract

*Objective:* The aim of the study was to determine the currently most frequent microbial findings in Bartholin's duct abscess.

*Methods:* Computerized records of microbial findings of 249 cases of Bartholin's duct abscess were retrospectively studied.

*Results:* In 129 cases, only 1 microbe and, in 117 cases, >1 microbe were recovered. In 3 cases, the flora was recorded as normal for the lower genital tract. Of all bacteria isolated, 252 were aerobic or facultative and 108 were anaerobic or microaerophilic. Aerobic or facultative bacteria alone caused 142 (57%) of the 249 cases, *Escherichia coli* being the most frequent isolate in this group. Anaerobic or microaerophilic bacteria alone caused 33 cases (13%), *Bacteroides* species and *Prevotella* species being most frequently identified. Both aerobic or facultative and anaerobic or microaerophilic bacteria were isolated in 70 cases (28%). *Candida albicans* alone caused 1 case of Bartholin's duct abscess. The sexually transmitted pathogens *Neisseria gonorrhoeae* and *Chlamydia trachomatis* were both involved in only 2 cases.

*Conclusions:* Bartholin's duct abscess was mainly caused by opportunistic bacteria, and sexually transmitted pathogens were only rarely involved in its pathogenesis. Since potentially pathogenic bacterial species were also frequently isolated, the use of antibiotics to complement the surgical treatment of Bartholin's duct abscess seems advisable, especially in patients with systemic symptoms.


Infectious Diseases in Obstetrics and Gynecology 1:265–268 (1994)
© 1994 Wiley-Liss, Inc.

### Microbiology of Bartholin's Duct Abscess

Anu Mattila, Ari Miettinen, and Pentti K. Heinonen

*Departments of Clinical Microbiology (A.M., A.M.) and Obstetrics and Gynecology (P.K.H.), University Hospital, and Department of Clinical Medicine (P.K.H.), University of Tampere, Tampere, Finland*

### Abstract

***Objective:*** **The aim of the study was to determine the currently most frequent microbial findings in Bartholin's duct abscess.**

***Methods:*** **Computerized records of microbial findings of 249 cases of Bartholin's duct abscess were retrospectively studied.**

***Results:*** **In 129 cases, only 1 microbe and, in 117 cases, >1 microbe were recovered. In 3 cases, the flora was recorded as normal for the lower genital tract. Of all bacteria isolated, 252 were aerobic or facultative and 108 were anaerobic or microaerophilic. Aerobic or facultative bacteria alone caused 142 (57%) of the 249 cases, *Escherichia coli* being the most frequent isolate in this group. Anaerobic or microaerophilic bacteria alone caused 33 cases (13%), *Bacteroides* species and *Prevotella* species being most frequently identified. Both aerobic or facultative and anaerobic or microaerophilic bacteria were isolated in 70 cases (28%). *Candida albicans* alone caused 1 case of Bartholin's duct abscess. The sexually transmitted pathogens *Neisseria gonorrhoeae* and *Chlamydia trachomatis* were both involved in only 2 cases.**

***Conclusions:*** **Bartholin's duct abscess was mainly caused by opportunistic bacteria, and sexually transmitted pathogens were only rarely involved in its pathogenesis. Since potentially pathogenic bacterial species were also frequently isolated, the use of antibiotics to complement the surgical treatment of Bartholin's duct abscess seems advisable, especially in patients with systemic symptoms.** © 1994 Wiley-Liss, Inc.

Key words
Bartholinitis, bacterial etiology, anaerobic bacteria

Bartholinitis is a common infection and involves populations far beyond the groups at risk for sexually transmitted diseases. In acute Bartholin's duct abscess, incision and drainage are considered the primary treatment. However, antibiotics are frequently included in the treatment in addition to surgical procedures, and the regimen is usually chosen based on empiric knowledge.

Previous studies on the bacteriology of Bartholin's duct abscess have emphasized the significance of gonococcus.[1–4] It has been reported to be involved in approximately ⅓ or more of cases.[1–4] Anaerobic bacteria have also been reported to be often involved.[2,5,6] During the last decade, *Chlamydia trachomatis* has been recognized as an important genital pathogen, and it has also been identified in Bartholin's duct abscess.[3,7] However, there are only a few reports on *C. trachomatis* causing bartholinitis,[3,7,8] and the incidence of chlamydial bartholinitis has not been thoroughly studied. Unusual etiologic agents causing bartholinitis, and even septic shock arising from Bartholin's duct abscess, have also been reported.[9–11]

The aim of this study was to determine which microbes are currently the most common pathogens in Bartholin's abscess so that the antibiotic regimens

**Address correspondence/reprint requests to Dr. Anu Mattila, Department of Clinical Microbiology, Tampere University Hospital, P.O. Box 2000, FIN-33521 Tampere, Finland.**

Received February 24, 1994
Accepted April 27, 1994

Clinical Study could be correctly directed against the most liable pathogens even before definite identification of the causing agent.

### SUBJECTS AND METHODS

Tampere University Hospital, Tampere, Finland, has kept computerized records of its microbial laboratory findings since 1969. To see which microbes are the most significant in Bartholin's duct abscess at the moment, we studied the records retrospectively from the beginning of 1983 to the end of 1989. During that time, the microbiological sampling and laboratory techniques followed an identical scheme.

Positive microbial findings were recorded from 220 women with bartholinitis during this time, 24 of whom suffered from 1 or more recurrences. Altogether, positive microbiological findings were available from 249 cases of Bartholin's duct abscess.

The abscesses were defined on a clinical basis. The samples were taken and studied according to general principles of diagnostic laboratory methods. The surface of the abscess was thoroughly cleansed with povidone-iodine and 70% alcohol. The content of the abscess was aspirated percutaneously or at the time of incision of the abscess with a needle attached to a plastic syringe of 0.5–20 ml. A Gram-stained smear was also made from each aspirate. If the interval between the aspiration of the abscess and inoculation of the cultures was fewer than 2 h, the syringe was capped and carried to the laboratory. If the interval between sampling and inoculation was longer than 2 h, the aspirate was injected into a transport medium for anaerobic samples (Portagerm®, BioMèrieux, Charbonnières-les-Bains Cedex, France). Alternatively, purulent material was collected on cotton-wool or dacron swabs and transported in Stuart tubes. Specimens for the culture of *C. trachomatis* were instantly placed into the transport medium, refrigerated at +4°C, and transported to the laboratory in 24 h. If the specimens for *C. trachomatis* could not be cultured in 24 h, they were stored deep frozen at −70°C until cultured.

Swabs or aliquots of each aspirate were inoculated onto chocolate agar, Columbia agar containing 7% horse blood, and into a thioglycollate tube, which in addition to thioglycollate medium also contained vitamin $K_1$ (0.1 μg/ml), $NaHCO_3$ (1 mg/ml), and 5% Fildes enrichment. These cultures were incubated at +37°C for 48 h and examined for identifying aerobic and facultative organisms.

For the isolation of anaerobic organisms, swabs or aliquots of each aspirate were inoculated onto fastidious anaerobe agar containing 5% horse blood. These cultures were incubated at +37°C for 48 h in an anaerobic jar with an atmosphere of 80% nitrogen, 10% carbon dioxide, and 10% hydrogen.

Specimens for the culture of *C. trachomatis* were inoculated on cycloheximide-treated McCoy cells. These cultures were incubated for 3 days at +35°C in an atmosphere containing 5% carbon dioxide, stained with iodine, and examined with a light microscope.

### RESULTS

Of all bacterial isolates recorded, 252 were aerobic or facultative and 108 were anaerobic or microaerophilic bacteria (Table 1). *Candida albicans* was identified in 3 cases, and mixed microbial flora referred to as normal for the lower genital tract mucosa in 3 cases. With or without other specific isolates recorded, mixed microbial flora, i.e., positive growth of several bacteria without any specific species that could be pointed out as the major agent, was recorded in 45 cases.

In 129 cases, only 1 microbe was recorded. In 111 cases, the sole pathogen was an aerobic or a facultative microbe and, in 17 cases, an anaerobic or a microaerophilic microbe. In 1 case, the sole pathogen was *C. albicans*. In 117 cases, >1 microbes were found. On the average, 1.65 bacteria/case were recorded, ranging from 1 to 7. Altogether, 45 different bacterial species were found (Table 1). Aerobic or facultative species alone caused 142 (57%) of the 249 cases. Anaerobic or microaerophilic species alone caused 33 cases (13%) of Bartholin's duct abscess. Both aerobic or facultative and anaerobic or microaerophilic species were found in 70 cases (28%). *C. albicans* was the only pathogenic agent in 1 case, along with aerobic or facultative agents in 1 case and both aerobic or facultative and anaerobic or microaerophilic agents in 1 case. In 3 cases, the flora was referred to as normal for the lower genital tract mucosa.

Most of the aerobic or facultative bacteria could be regarded as being opportunistic pathogens. The most frequently isolated microbe representing this group was *Escherichia coli*. The sexually transmit-

**TABLE 1. Bacterial findings in 249 Bartholin's abscess cases of 220 patients**

| | N |
|---|---|
| *Aerobic/Facultative Bacteria; N = 252* | |
| Gram-negative | |
| *Escherichia coli* | 77 |
| *Proteus* group[a] | 9 |
| *Haemophilus influenzae* | 4 |
| *Klebsiella pneumoniae* | 4 |
| *Klebsiella oxytoca* | 1 |
| *Salmonella enteritidis* | 1 |
| Gram-positive | |
| *Staphylococcus* species | 58 |
| *Streptococcus agalactiae* | 19 |
| *Enterococcus faecalis* | 18 |
| *Streptococcus alfa-haemolyticus* | 15 |
| *Staphylococcus aureus* | 13 |
| *Streptococcus beta-haemolyticus*[b] | 8 |
| *Streptococcus milleri* | 6 |
| *Streptococcus non-haemolyticus* | 5 |
| *Diplococcus pneumoniae* | 4 |
| *Corynebacterium* species | 1 |
| *Micrococcus* species | 1 |
| *Staphylococcus saprophyticus* | 1 |
| *Streptococcus equinis* | 1 |
| *Streptococcus mitis* | 1 |
| *Streptococcus* species | 1 |
| Sexually transmitted pathogens | |
| *Chlamydia trachomatis* | 2 |
| *Neisseria gonorrhoeae* | 2 |
| *Anaerobic/Microaerophilic Bacteria; N = 108* | |
| Gram-negative | |
| *Prevotella* species[c] | 23 |
| *Bacteroides* species | 17 |
| *Bacteroides fragilis* group | 10 |
| *Fusobacterium* species | 3 |
| *Propionibacterium* species | 2 |
| *Veillonella* species[d] | 2 |
| *Gardnerella vaginalis* | 1 |
| *Porphyromonas asaccharolytica* | 1 |
| Gram-positive | |
| *Peptostreptococcus* species | 21 |
| *Peptococcus* species [e] | 19 |
| *Lactobacillus* species | 5 |
| Microaerophilic *streptococcus* | 4 |

[a]*Proteus* group (N = 9) includes here *P. mirabilis* (N = 7) and *Morganella morgani* (N = 2).
[b]*Streptococcus beta-haemolyticus* (N = 7) includes here *S. beta-haemolyticus* group G (N = 4), *S. beta-haemolyticus* group F (N = 2), *S. beta-haemolyticus* group A (N = 1), and *S. beta-haemolyticus* group C (N = 1).
[c]*Prevotella* species (N = 23) includes here *P. bivia* (N = 16), *P. melaninogenica* (N = 4), *P. intermedia* (N = 2), and *P. oris* (N = 1).
[d]*Veillonella* species (N = 2) includes here *V. parvula* (N = 1), and anaerobic gram-negative coccus (N = 1).
[e]*Peptococcus* species (N = 19) includes here *Peptococcus* species (N = 9), *P. asaccharolyticus* (N = 8), and anaerobic coccus (N = 2).

ted pathogens *Neisseria gonorrhoeae* and *Chlamydia trachomatis* were not found in significant numbers. Of the aerobic or facultative bacteria regarded as being commensals, the most frequent ones were coagulase-negative *Staphylococcus* species. Of the anaerobic or microaerophilic bacteria, the most frequent isolates were *Bacteroides* and *Prevotella* species.

### DISCUSSION

The significance of polymicrobial infections with anaerobic bacteria involved has been documented in severe gynecological infections with abscess formation, such as pelvic inflammatory disease with tubo-ovarian abscess.[12] In contrast, albeit frequently isolated in conjunction with less severe gynecological infections, such as endometritis, the pathogenic role of anaerobic bacteria in such infections remains questionable. As a numerous part of vaginal flora, anaerobic bacteria often contaminate specimens taken vaginally, which makes it difficult to assess their pathogenic significance. With regard to bartholinitis, the contamination of specimens with vaginal flora is also possible, albeit less likely. In our study, anaerobic or microaerophilic bacteria were involved in 103 (41%) of the 249 cases with bartholinitis either alone or in conjunction with aerobic or facultative bacteria. *Bacteroides* and *Prevotella* species were the most common anaerobic bacteria isolated, which is an accordance with previous studies on the subject.[5,6] According to the current results, the use of antibiotic regimens covering anaerobic bacteria should be advantageous in the treatment of acute bartholinitis.

*E. coli* was the most frequent facultative microbe causing Bartholin's duct abscess in our study. In addition to its role as the major cause of urinary tract infections, *E. coli* has been reported as an important cause of various infections in the female genital tract, including bartholinitis.[2,5,6,13–15] On the other hand, the pathogenic role of aerobic or facultative bacteria regarded as being commensals is questionable. The possibility of their being contaminants from mucosal surfaces cannot be excluded.

The incidence of gonococcal infection has decreased markedly in Scandinavian countries since the 1970s. Therefore, it is not surprising that the gonococcus is no longer a major pathogen in Bartholin's duct abscess in this population. In contrast, although the incidence of *C. trachomatis* has increased during the last decade, our study shows that *C. trachomatis* is of no special significance in causing Bartholin's duct abscess. *C. trachomatis* should be respected as a rare cause of bartholinitis and it is likely that the Bartholin's gland is not the primary site for chlamydial infection.

The recommended treatment of Bartholin's duct abscess is incision and drainage. Opinions on the benefit of including antibiotics in the treatment are somewhat discordant. In our study, a considerable number of Bartholin's duct abscess cases were caused by bacteria regarded as being potential pathogens, some of them documented to cause even septic shock arising from bartholinitis, e.g., *E. coli* and *Streptococcus beta-haemolyticus* group A.[10,11] It would seem advisable to include antibiotics to avoid the spread of infection in the treatment of Bartholin's duct abscess in addition to surgical procedures, especially in patients presenting with systemic symptoms. The possibility of a sexually transmitted infection as a rare cause of Bartholin's duct abscess emphasizes the importance of obtaining bacterial and chlamydial specimens as a part of the routine treatment.

### REFERENCES

1. Rees E: Gonococcal bartholinitis. Br J Vener Dis 43:150–156, 1967.
2. Lee Y-H, Rankin JS, Alpert S, Daly AK, McCormack WM: Microbiological investigation of Bartholin's gland abscesses and cysts. Am J Obstet Gynecol 129:150–154, 1977.
3. Davies JA, Rees E, Hobson D, Karayiannis P: Isolation of *Chlamydia trachomatis* from Bartholin's ducts. Br J Vener Dis 54:409–413, 1978.
4. Cheetham DR: Bartholin's cyst: Marsupialization or aspiration? Am J Obstet Gynecol 152:569–570, 1985.
5. Wren MWD: Bacteriological findings in cultures of clinical material from Bartholin's abscess. J Clin Pathol 30: 1025–1027, 1977.
6. Brook I: Aerobic and anaerobic microbiology of Bartholin's abscess. Surg Obstet Gynecol 169:32–34, 1989.
7. Saul HM, Grossman MB: The role of *Chlamydia trachomatis* in Bartholin's abscess. Am J Obstet Gynecol 158:576–577, 1988.
8. Bleker OP, Smalbraak DJC, Schutte MF: Bartholin's abscess: The role of *Chlamydia trachomatis*. Genitourin Med 66:24–25, 1990.
9. Morton BD, McCarthy LR: Bartholinitis—An unusual etiologic agent. Obstet Gynecol 55:97S–98S, 1980.
10. Carson GD, Smith LP: *Escherichia coli* endotoxic shock complicating Bartholin's gland abscess. CMA J 122: 1397–1398, 1980.
11. Shearin RS, Boehlke J, Karanth S: Toxic shock-like syndrome associated with Bartholin's gland abscess: Case report. Am J Obstet Gynecol 160:1073–1074, 1989.
12. Eschenbach DA, Buchanan TM, Pollock HM, et al.: Polymicrobial etiology of acute pelvic inflammatory disease. N Engl J Med 293:166–171, 1975.
13. Heinonen PK: Carbon dioxide laser in the treatment of abscess and cyst of Bartholin's gland. J Obstet Gynecol 10:535–537, 1990.
14. Brihmer C, Kallings I, Nord C-E, Brundin J: Salpingitis; aspects of diagnosis and etiology: A 4-year study from a Swedish capital hospital. Eur J Obstet Gynecol Reprod Biol 24:211–220, 1987.
15. Sweet RL, Draper DL, Schachter J, James J, Hadley WK, Brooks GF: Microbiology and pathogenesis of acute salpingitis as determined by laparoscopy: What is the appropriate site to sample. Am J Obstet Gynecol 138: 985–989, 1980.
